# Bioavailability of *n*-3 fatty acids from *n*-3-enriched foods and fish oil with different oxidative quality in healthy human subjects: a randomised single-meal cross-over study

**DOI:** 10.1017/jns.2016.34

**Published:** 2016-10-28

**Authors:** Inger Ottestad, Berit Nordvi, Gjermund Vogt, Marianne Holck, Bente Halvorsen, Kirsti W. Brønner, Kjetil Retterstøl, Kirsten B. Holven, Astrid Nilsson, Stine M. Ulven

**Affiliations:** 1 Department of Health, Nutrition and Management, Faculty of Health Sciences, Oslo and Akershus University College of Applied Sciences, PO Box 4 St. Olavs plass, 0130 Oslo, Norway; 2 Department of Nutrition, Institute for Basic Medical Sciences, University of Oslo, PO Box 1046 Blindern, 0317 Oslo, Norway; 3 Faculty of Medicine, University of Oslo, Oslo, Norway; 4 TINE SA, R&D Centre, PO Box 7 Kalbakken, 0902 Oslo, Norway; 5 Eurofins Food & Feed Testing Norway AS, Møllebakken 40, 1538 Moss, Norway; 6 Research Institute of Internal Medicine, Oslo University Hospital, Rikshospitalet, PO Box 4950 Nydalen, 0424 Oslo, Norway; 7 Lipid Clinic, Medical Department, Oslo University Hospital, Rikshospitalet, PO Box 4950 Nydalen, 0424 Oslo, Norway; 8 Department of Endocrinology, Morbid Obesity and Preventive Medicine, Norwegian National Advisory Unit on Familial Hypercholesterolemia, Oslo University Hospital, Rikshospitalet, PO Box 4950 Nydalen, 0424 Oslo, Norway; 9 Nofima AS, Norwegian Institute of Food, Fisheries and Aquaculture Research, Osloveien 1, 1430 Ås, Norway

**Keywords:** Fish oil, Oxidised fish oil, *n*-3-Enriched food, Cross-over studies, EPA, DHA, Postprandial TAG, LC *n*-3 FA, long-chain *n*-3 fatty acid

## Abstract

Regular consumption of long-chain *n*-3 fatty acids (LC *n*-3 FA) reduces postprandial triacylglycerolaemia. Functional foods and supplements are alternative sources of LC *n*-3 FA; however, emulsification technologies, food matrices and altered lipid oxidation levels affect their bioavailability. Moreover, which functional foods are optimal LC *n*-3 FA carriers is unknown. The aim of the study was to determine the bioavailability of LC *n*-3 FA and the postprandial TAG response after the intake of oxidised or non-oxidised cod liver oil and after the intake of emulsified or non-emulsified LC *n*-3 FA using novel functional food items as LC *n*-3 FA carriers in a randomised cross-over acute study. A total of twenty-four healthy subjects completed the study in which subjects consumed one of four different test meals containing 1·5 g LC *n*-3 FA, or a control meal with no LC *n*-3 FA. Postprandial TAG-rich lipoproteins were isolated and their fatty acid composition was measured. The LC *n*-3 FA from emulsified foods were more rapidly incorporated into TAG-rich lipoproteins compared with non-emulsified foods. The incorporation of LC *n*-3 FA was similar for oils emulsified in yogurt or juice and was unaffected by the oxidative status of the oil. Postprandial TAG levels did not differ among the various test meals. In conclusion, emulsification increases the bioavailability of LC *n*-3 FA through a more rapid incorporation into TAG-rich lipoproteins, and juice and yogurt are equally suited as LC *n*-3 FA carriers. The acute intake of oxidised cod liver oil does not influence the incorporation of LC *n*-3 FA into TAG-rich lipoproteins.

Regular consumption of fish and fish oil supplements containing long-chain *n*-3 fatty acids (LC *n*-3 FA) reduces fasting and postprandial TAG levels^(^^1^^–^^3^^)^. It is well known that postprandial hypertriacylglycerolaemia significantly affects the development and progression of atherosclerosis^(^^4^^–^^6^^)^. In addition, postprandial hypertriacylglycerolaemia is an independent risk factor for adverse cardiovascular events and death^(^^7^^–^^9^^)^.

Dietary sources of LC *n*-3 FA are scarce and the food industry uses different food technologies, such as emulsification, to incorporate LC *n*-3 FA into a variety of novel functional foods to increase their availability to consumers. Studies have shown that the bioavailability (digestion and absorption) of LC *n*-3 FA is affected by the chemical forms of LC *n*-3 FA (i.e. TAG, phospholipids, ethyl esters), by emulsification and by the concomitant intake of foods, especially fat (the food matrix effect)^(^^10^^–^^12^^)^. It is still unclear which food items are best suited as LC *n*-3 FA carriers, in terms of providing optimal bioavailability of LC *n*-3 FA and a postprandial TAG response.

LC *n*-3 FA are highly susceptible to lipid oxidation^(^^13^^)^, but no international legislation standard for fish oil quality, defined as maximum oxidation level, has been established for fish oil used for human consumption^(^^14^^,^^15^^)^. Recent studies have reported elevated peroxide levels in commercialised fish oil capsules, which have led to some concern that regular consumption of oxidised marine oils may negatively affect human health^(^^16^^–^^21^^)^. In a previous study, we did not observe any changes in fasting plasma LC *n*-3 FA levels, lipids or several other oxidative stress markers after 7 weeks of consumption of oxidised compared with non-oxidised fish oil^(^^22^^)^. However, another study reported that the intake of less oxidised *n*-3 FA supplements for 30 d reduced blood lipids and blood pressure compared with highly oxidised LC *n*-3 FA supplements^(^^23^^)^. Postprandial studies have also shown that the intake of oxidised compared with non-oxidised vegetable oils increased lipid peroxides in chylomicrons^(^^24^^–^^26^^)^. Lipid oxidation products are highly reactive and can form stable covalent adducts with macromolecules such as proteins and lipids. Thus, the absorption of fatty acids and the enzymes involved in the lipoprotein assembly could potentially be affected by intake of oxidised lipids^(^^27^^,^^28^^)^. Whether consumption of oxidised *v*. non-oxidised fish oil alters the bioavailability of LC *n*-3 FA has not been previously investigated in human subjects.

The aims of the present study were to investigate the bioavailability of LC *n*-3 FA and the postprandial TAG response after intake of oxidised *v*. non-oxidised cod liver oil and after the intake of equal amounts of emulsified *v*. non-emulsified LC *n*-3 FA in healthy human subjects using novel functional food items as carriers of LC *n*-3 FA (yogurt *v*. juice).

## Materials and methods

### Subjects

Healthy non-smoking men and women aged 20–50 years (*n* 32) were recruited from the Akershus University College, Norway. Subjects that had a fish consumption ≥ two servings per week or used cod liver oil/fish oil supplements >1/week were excluded. Other exclusion criteria included chronic diseases and diseases known to interfere with lipid absorption such as lactose intolerance and other malabsorption diseases. In addition, subjects with circulating levels of total cholesterol >7·5, TAG >4 mmol/l, C-reactive protein ≥10 mmol/l and thyroid-stimulating hormone, triiodothyronine and thyroxine above or below the normal reference ranges were excluded. Furthermore, hypertension (≥160/100 mmHg), BMI ≥35 kg/m^2^, change in body weight during the last 3 months (weight ±5 %), pregnancy and lactation were also used as exclusion criteria. The use of lipid-lowering and antihypertensive medications, and the use of dietary supplements known to interfere with plasma cholesterol such as soluble fibre and plant sterols were not permitted during the study period. The study was approved by the Regional Committee of Medical Ethics (approval no. 1.2007.2870) and by the Norwegian Social Science Data Services (approval no. 18790). Written informed consent for participation was obtained from each participant and the study complied with the Declaration of Helsinki.

### Study design

The study was a randomised five-period cross-over study. The participants were randomised into five groups each receiving a test meal every second week. In total, all participants received the five test meals. Each test meal consisted of similar amounts of yogurt (203 g), juice (324 g), bread (50 g), butter (3·2 g) and an oil shot (7·9 g). The test meals contained 1·5 g EPA+ DHA from either (A) emulsified LC *n*-3 FA in yogurt, (B) emulsified LC *n*-3 FA in juice, (C) non-emulsified, non-oxidised LC *n*-3 FA as an oil shot or (D) non-emulsified, oxidised LC *n*-3 FA as an oil shot, or (E) a reference test meal containing no LC *n*-3 FA. All test meals were prepared with different types of oils, which are further outlined in Table 1. The energy content, and the percentage of energy (E %) from carbohydrates (43·9 E %), proteins (7·7 E %) and total fat (48·4 E %) were equal in all test meals. The composition of the fatty acids in the test meals is shown in Supplementary Table S1.

**Table 1.** Type of oil added to the five test meals

		Test meal				
		A	B	C	D	E
Food item	Oil type	Yogurt meal	Juice meal	Oxidised cod liver oil meal	Non-oxidised cod liver oil meal	Reference meal
Yogurt, emulsified	Cod liver	X	–	–	–	–
	High-oleic sunflower	–	X	X	X	X
Juice, emulsified	Cod liver	–	X	–	–	–
	High-oleic sunflower	X	–	X	X	X
Oil shot	Cod liver	–	–	X*	X*	–
	High-oleic sunflower	X	X	–	–	X

PV, peroxide value; AV, anisidine value.

* The cod liver oil was oxidised (PV 16 meq/kg; AV 11) in test meal C, and non-oxidised (PV 1·5 meq/kg; AV 2·1) in test meal D, respectively. Yogurt and juice were emulsified with cod liver oil or high-oleic sunflower oil to make test and control food items, respectively.

**Fig. 1.**
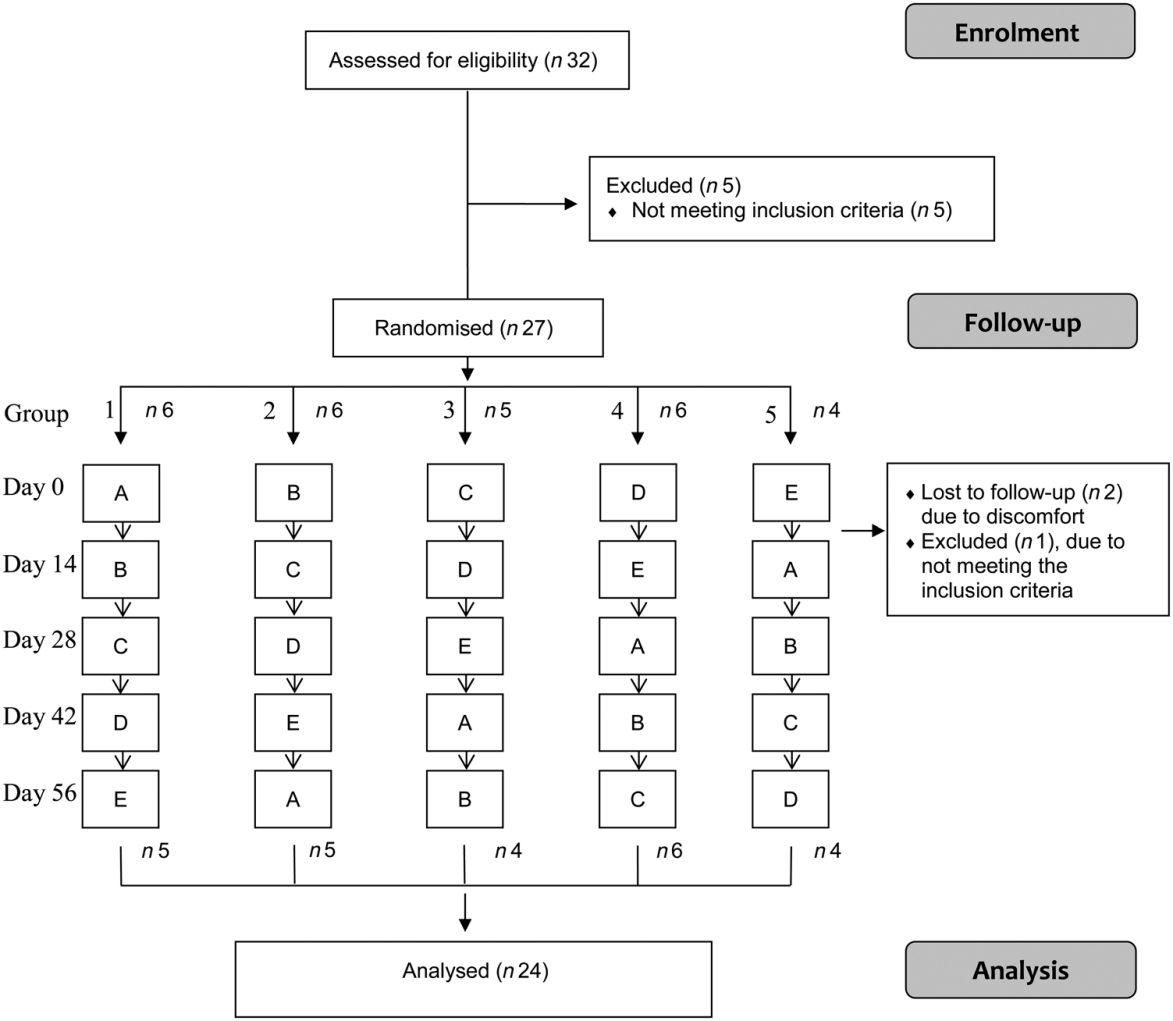
Flow diagram showing the flow of participants through the trial. The participants were randomised into five groups in which the five test meals (A, B, C, D and E) were consumed in a predetermined sequence. Two participants were lost to follow-up after day 0 due to discomfort (one in group 1 and one in group 2), and one was excluded after day 0 due to not meeting the inclusion criteria (group 3).

Blocked randomisation was used to divide the subjects into five groups, and the participants received the test meals in one of the five fixed orders (group 1: ABCDE, group 2: BCDEA, group 3: CDEAB, group 4: DEABC, group 5: EABCD). The flow of subjects through the study is outlined in Fig. 1. The study was blinded for the participants and all study investigators involved, except for those preparing and serving the test meals. During the study period, the subjects were encouraged to have a stable diet and a fish intake as reported at inclusion. The last 2 d prior to the test day, the subjects were not allowed to consume fish or other fish products containing marine LC *n*-3 FA. A dietitian provided information about which food products they should avoid. During the test day, the subjects were not allowed to use chewing tobacco, coffee, tea or chewing gum, but water was allowed *ad libitum* throughout the day. The test meals were eaten at the University College under observation, and all foods and beverage were consumed within 20 min.

### Preparation of test products

#### Preparation of emulsified long-chain *n*-3 oil in yogurt and juice

Yogurt and juice enriched with LC *n*-3 FA were produced by mixing commercial yogurt (162 g; 3·4 % fat) or juice (284 g; 0·2 % fat) with emulsions (40·5 g) containing functional food-grade cod liver oil (7·9 g) to make test foods with 1·5 g EPA + DHA. Matching control foods containing no LC *n*-3 FA were prepared by adding similar amounts of emulsion (40·5 g) with high-oleic sunflower oil (7·9 g) to the commercial yogurt or juice. The final fat content of the yogurt and juice was similar for test and control. The fat content and the macronutrient composition of the food items prepared and included in the test meals are outlined in Table 2. Food products were prepared and delivered by TINE SA, and the functional food-grade cod liver oil and the high-oleic sunflower oil were provided by TINE SA (*Gadidae* sp., EPADHA Oil 1200) and AarhusKarlshamn AB, respectively.

**Table 2.** Macronutrient composition of the food items used in the test meals

	Yogurt*		Juice*		Oil shot†		Butter		Bread	
	203 g/meal		324 g/meal		7·9 g/meal		3·2 g/meal		50 g/meal	
Macronutrients	g/100 g	g/food item	g/100 g	g/food item	g/100 g	g/food item	g/100 g	g/food item	g/100 g	g/food item
Carbohydrates	12·2	19·8	10·4	29·5	0·0	0·0	0·5	0·0	44·6	22·3
Protein	3·6	5·8	0·7	2·0	0·0	0·0	0·5	0·0	9·4	4·7
Fat	7·1	14·4	2·9	9·5	7·9	7·9	82·0	2·6	1·4	0·7

* The yogurt and juice meals were made by adding 40·5 g emulsion containing either cod liver oil or high-oleic sunflower oil to 162 g yogurt and 283·5 g juice.

† Macronutrient composition of non-oxidised, oxidised and high-oleic sunflower oil shots.

#### Preparation of oxidised cod liver oil

Functional food-grade cod liver oil (*Gadidae* sp.) was oxidised at Nofima Mat (Ås, Norway) as described elsewhere^(^^22^^)^. Briefly, the batch with high-quality non-oxidised oil was divided into two parts. The non-oxidised oil had peroxide value and anisidine value of 1·5 meq/kg and 2·1, respectively (measured according to AOCS Official Method Cd 8–53 and Cd 18–90, respectively). The oxidised oil was placed at room temperature and oxygenated until the oil was oxidised to a peroxide value of 16 meq/kg and an anisidine value of 11. The characterisation of the fish oils is outlined in Table 3.

**Table 3.** Characterisation of the oils

	Non-oxidised cod liver oil	Oxidised cod liver oil	High-oleic sunflower oil
Fatty acids			
SFA (g/100 g)	16	16	7
MUFA (g/100 g)	47	47	76
PUFA (g/100 g)	28	28	9
*n*-3 Fatty acids			
EPA (20 : 5*n*-3) (g/100 g)	9	9	0
DHA (22 : 6*n*-3) (g/100 g)	11	11	0
DPA (22 : 5*n*-3) (g/100 g)	1·0	1·0	0·0
ALA (18 : 3*n*-3) (g/100 g)	0·8	0·8	0·3
Oxidation level			
PV (meq/kg)	1·5	16	4
AV	2·1	11	3
Totox*	5·1	43	11

DPA, docosapentaenoic acid; ALA, α-linolenic acid; PV, peroxide value; AV, anisidine value.

* Totox = 2 × PV + AV.

### Blood samples

The day prior to blood sampling, the subjects were told to refrain from alcohol consumption and vigorous physical activity, and the subjects were instructed to consume a low-fat meal during the afternoon and evening. Venous blood samples were drawn after an overnight fast (≥12 h) and at 2, 4 and 6 h after consumption of the test meals. Serum was obtained from silica gel tubes (BD Vacutainer) and kept at room temperature for 30–120 min and then centrifuged at 1500 ***g*** for 12 min at 4°C. Serum total, LDL- and HDL-cholesterol, TAG, glucose, C-reactive protein, alanine transaminase (ALT), creatinine, thyroid-stimulating hormone, triiodothyronine and thyroxine were measured by standard methods at Fürst Medical Laboratory (Oslo, Norway). Plasma was obtained from heparin vacuum tubes (BD Vacutainer) and was immediately centrifuged at 1300 ***g*** for 10 min at 4°C. Plasma was then isolated, and sucrose was added to a final 0·6 % sucrose solution before freezing (−80°C). Samples were analysed for fatty composition in TAG-rich lipoproteins within 6 months of freezing.

### Lipoprotein isolation

TAG-rich lipoproteins; chylomicrons (CM) and VLDL (*d* 1·006 kg/l (g/ml), were isolated from plasma by sequential ultracentrifugation using a TI 80 rotor (Beckman Optima LE-80K ultracentrifuge), using soluble sodium bromide (NaBr) to adjust the final volume. Plasma was centrifuged at 570 000 ***g*** for 5 h and 15 min at 10°C, and the TAG-rich fraction (CM + VLDL) was harvested, N_2_ flushed, snap frozen and stored at −80°C until further analysis.

### Fatty acid analysis of TAG-rich lipoprotein fraction

Lipids in the TAG-rich lipoprotein fraction were extracted using the method of Bligh & Dyer^(^^29^^)^. The fatty acids in extracts and oils were transmethylated by a modified version of a method described by Mason and Waller. Briefly, 1 µl of the methyl ester solution was injected splitless on a GC (HP model no. G1530A) equipped with an autosampler (HP 6890 Series Injector) and flame ionization detector. The analytes were separated on a BPX70 column (0·25 mm internal diameter, 60 m, 0·25 µm film) from SGE with He as the carrier gas, using a temperature program of 70°C for 1 min, increasing by 30°C/min to 170°C, then by 1·5°C/min to 200°C and by 3°C/min to 220°C with a final hold time of 5 min. Peaks were integrated with HP GC ChemStation software (rev. B.0101; Agilent Technologies) and identified by use of external standards. CV were <5 %.

### Fatty acid analysis of food

Lipids in the foods were extracted using a modified method of Bligh & Dyer^(^^29^^)^. The fatty acids in extracts and oils were transmethylated and 1 µl of the methyl ester solutions was injected splitless on a GC (Agilent GC-6890N; Agilent Technologies) equipped with an autosampler (Agilent G-2614A) and flame ionization detector. The analytes were separated on a HP-88 column (0·25 mm internal diameter, 100 m, 0·20 µm film) from Agilent with He as the carrier gas, using a temperature program of 60°C for 10 min, increasing by 10°C/min to 170°C, and by 2·0°C/min to 216°C and with a final hold time of 35 min. Peaks were integrated with HP GC ChemStation software (rev. B.0101; Agilent Technologies) and identified by use of external standards. CV were <5 %.

### Statistical analysis

Sample size was calculated from a pilot study in which six subjects were included. To detect a 40 % difference in EPA (change from baseline between groups), using 80 % power and 0·05 level of significance (two-sided), a total of twenty subjects were suggested to be necessary. A 20 % dropout was expected; therefore a total of twenty-five subjects were included. Parametric statistics were used and data are presented as mean values with standard deviations or standard errors. Repeated ANOVA measurements with Bonferroni *post hoc* analysis were used to compare differences among the test meals at each time point. All analyses were performed using SPSS for Windows (version 20; SPSS, Inc.).

## Results

In all, twenty-four subjects (six men and eighteen women) aged 32 ± 8 years and with blood lipid levels within the normal range completed the study (Table 4).

**Table 4.** Baseline characteristics* (Mean values and 2 standard deviations)

	Mean	2 sd
Subjects (*n*)		
Male	6	
Female	18	
Age (years)	32	8
BMI (kg/m^2^)	24	3
Glucose (mmol/l)	4·7	0·5
Total cholesterol (mmo/l)	5·0	1·1
LDL-cholesterol (mmol/l)	2·8	0·9
HDL-cholesterol (mmol/l)	1·6	0·4
TAG (mmol/l)	0·8	0·4

* Data were collected at baseline before the first test day.

**Fig. 2.**
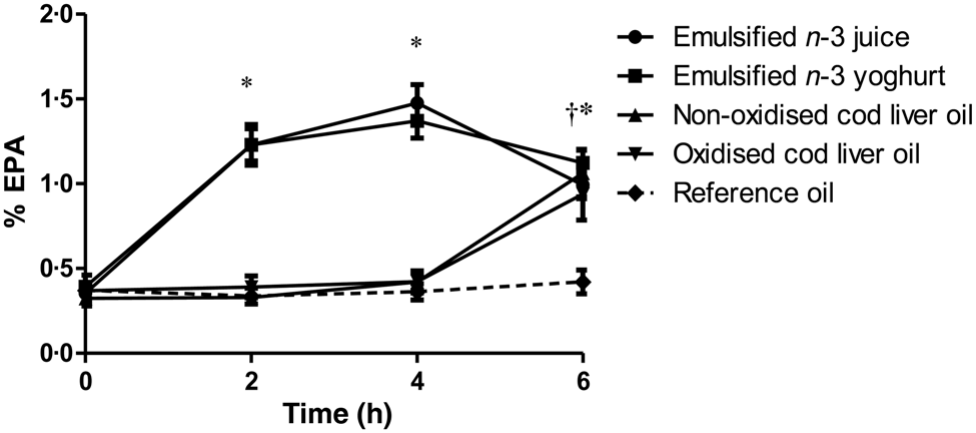
Percentage of EPA in total lipids from the TAG-rich lipoprotein fraction (chylomicrons/VLDL) at baseline and after 2, 4 and 6 h after intake of test meals containing 1·5 g EPA + DHA in either emulsified juice or yogurt, non-oxidised cod liver oil, oxidised cod liver oil or reference oil lacking EPA or DHA. Values are means, with standard errors represented by vertical bars. *P < 0·001 for juice and yogurt compared with baseline. † *P* = 0·007 for non-oxidised cod liver oil and *P* < 0·001 for oxidised cod liver oil compared with baseline.

**Fig. 3.**
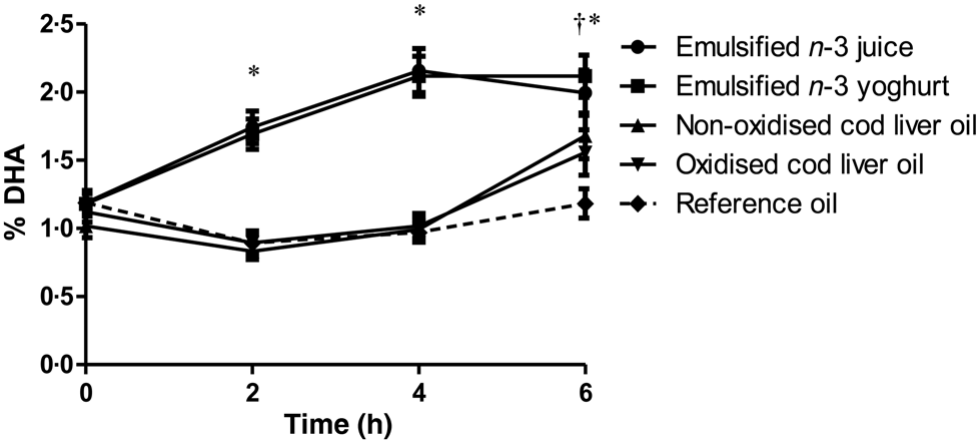
Percentage of DHA in total lipids from the TAG-rich lipoprotein fraction (chylomicrons/VLDL) at baseline and after 2, 4 and 6 h after intake of test meals containing 1·5 g EPA + DHA in either emulsified juice or yogurt, non-oxidised cod liver oil, oxidised cod liver oil or reference oil lacking EPA or DHA. Values are means, with standard errors represented by vertical bars. *P < 0·001 for juice and yogurt compared with baseline. † *P* < 0·001 for non-oxidised and oxidised cod liver oil compared with baseline.

The postprandial concentrations of EPA and DHA in TAG-rich lipoproteins were significantly different after the intake of test meals where LC *n*-3 FA were emulsified in juice or yogurt, compared with the intake of test meals where the LC *n*-3 FA were given as non-emulsified cod liver oil (Figs 2 and 3). Compared with baseline, the levels of EPA and DHA in TAG-rich lipoproteins were significantly increased after 2 and 4 h and reached a maximum level 4 h after consumption of test meals containing LC *n*-3 FA-enriched juice and yogurt (*P* < 0·001 for all comparisons). No significant differences between juice and yogurt were observed for EPA or DHA levels at any time point. Despite approximately similar content of EPA (9 %) and DHA (11 %) in the fish oil, the relative increase of EPA was higher compared with that of DHA, at 2 and 4 h after intake (Figs 2 and 3). EPA levels decreased significantly between 4 and 6 h after intake of emulsified juice and yogurt (*P* = 0·001 and *P* < 0·001, respectively); however, DHA levels remained unchanged between 4 and 6 h (*P* = 0·141 and *P* = 0·676, respectively). Similar time–response curves for EPA and DHA levels in TAG-rich lipoproteins after the intake of test meals containing non-emulsified cod liver oil shots (oxidised and non-oxidised) were observed. Compared with baseline, a significant increase in both EPA and DHA levels were only observed after 6 h (*P* < 0·001 for all comparisons) (Figs 2 and 3). No significant differences in EPA or DHA levels were observed between the intake of oxidised and non-oxidised cod liver oil at any of the time points.

When comparing all test meals, significantly higher EPA and DHA levels were observed at 2 and 4 h after intake of LC *n*-3 FA-enriched juice and yogurt, compared with cod liver oil shots (oxidised or non-oxidised; *P* < 0·001 for all comparisons). At 6 h, the EPA levels were significantly increased compared with the reference meal (*P* < 0·001 for all comparisons), but they were similar among the different test meals. However, 6 h after intake, the levels of DHA were significantly higher after consumption of LC *n*-3 FA-enriched yogurt and juice, compared with that of oxidised cod liver oil (*P* = 0·007 and *P* = 0·012, respectively). In addition, a significantly higher DHA level was observed 6 h after of LC *n*-3 FA-enriched yogurt, but not juice, compared with the intake of non-oxidised cod liver oil (*P* = 0·048).

No significant differences in serum TAG levels were observed among the five test meals at baseline or postprandially at any time point. For all test meals, the postprandial serum TAG levels were significantly increased after 2 h (within-group analysis *P* < 0·001 for all test meals) and 4 h (for all test meals; *P* < 0·05); the maximum level was reached 2 h after intake (Table 5). Correlation analysis for each of the test meals was performed to determine if fasting TAG levels were associated with serum postprandial TAG levels. No correlation between fasting TAG levels (mmol/l) and the relative increase in TAG levels (%) was observed among the different test meals at any time point (data not shown). Finally, no correlations were observed between EPA or DHA levels in TAG lipoproteins and the postprandial increase in serum TAG (%) at any time point (data not shown).

**Table 5.** Serum TAG (mmol/l) before and after intake of each test meal (Mean values and standard deviations)

Time…	0 h		2 h		4 h		6 h	
Test meal	Mean	sd	Mean	sd	Mean	sd	Mean	sd
Emulsified *n*-3 juice	0·8	0·5	1·4**	0·8	1·1*	0·8	0·9	0·6
Emulsified *n*-3 yogurt	0·8	0·5	1·4**	0·9	1·2*	1·0	0·9	0·7
Oxidised cod liver oil	0·9	0·4	1·5**	0·8	1·2*	0·8	1·0	0·6
Non-oxidised cod liver oil	0·9	0·5	1·5**	0·9	1·3*	0·9	1·0	0·7
Reference oil	0·8	0·4	1·4**	0·7	1·3*	0·8	1·0	0·6

Mean value was significantly different from that at baseline: * *P* < 0·05, ** *P* < 0·001.

## Discussion

The present study shows that EPA and DHA from food items containing emulsified LC *n*-3 FA are more quickly incorporated into TAG-rich lipoproteins compared with non-emulsified LC *n*-3 FA. Neither the oxidative status (oxidised *v*. non-oxidised oils) nor the food matrix (LC *n*-3 FA-enriched juice or yogurt) influenced the incorporation of LC *n*-3 FA into TAG-rich lipoproteins. The postprandial rise in serum TAG did not differ among the test meals.

Several long-term human studies have shown that the bioavailability EPA and DHA from fish oil capsules is influenced by the chemical form of LC *n*-3 FA^(^^10^^–^^12^^,^^30^^)^. In line with our results, other acute studies have reported a more rapid absorption from food items with emulsified LC *n*-3 FA^(^^31^^–^^35^^)^. One possible explanation is that emulsified oils bypass the normal physiological digestion process, in which fat globules are broken down into smaller emulsion droplets^(^^32^^)^. Another possibility is that emulsification of the oil reduces droplet size, causing them to be more dispersed, which leads to increased pancreatic lipase activity^(^^36^^)^. Thus, increased pancreatic lipase activity may explain, at least in part, the rapid incorporation of EPA and DHA into TAG-rich lipoproteins after intake of food items containing emulsified LC *n*-3 FA. Emulsification of marine oils improves palatability and tolerance (remove ‘fishy flavour’), making the oils suitable for fortified food production. However, the food matrix (the food texture and the fat content of the food) may affect the digestion and the incorporation of LC *n*-3 FA in chylomicrons. In contrast to our study, a more rapid absorption of LC *n*-3 FA after intake of yogurt was observed when the same amount of emulsified fish oil was added to either yogurt or a fitness bar^(^^31^^)^. The authors suggested that the solid food matrix of the fitness bar prevented the release of LC *n*-3 FA during digestion^(^^31^^)^. The semi-liquid of yoghurt and liquid form of juice may explain the similar and rapid release of LC *n*-3 FA observed in the present study.

LC *n*-3 FA from oxidised compared with non-oxidised oils were similarly incorporated into TAG-rich lipoproteins. This result is in line with our previous study where the plasma levels of EPA and DHA were similar after 7 weeks of intake of oxidised compared with non-oxidised cod liver oil capsules^(^^22^^)^. Despite differences in lipid oxidation products in the oxidised and non-oxidised cod liver (measured by peroxide value and anisidine value; Table 1), the concentration of EPA and DHA was equal in the oils. Thus, the level of oxidation of cod liver oil did not affect the incorporation rates of EPA and DHA into TAG-rich lipoproteins.

We did not observe any effect of LC *n*-3 FA intake on postprandial TAG levels. Studies in normo- and hypertriacylglycerolaemic subjects have shown that regular consumption of LC *n*-3 FA reduces postprandial TAG levels^(^^37^^–^^39^^)^; however, decreases in postprandial TAG levels have not been observed in acute studies of normotriacylglycerolaemic subjects^(^^3^^,^^31^^,^^40^^)^. The stable fish consumption (≤2 meals/week) among some of the participants over the last 4 months prior to inclusion and throughout the study period may have influenced the postprandial TAG results in the present study. Moreover, we cannot rule out that the results would have been different in a study group with a lower baseline consumption of LC *n*-3 FA. A limitation of our study is that the absolute availability of LC *n*-3 FA could not be compared because we did not take blood samples beyond 6 h.

In conclusion, the present study demonstrates that emulsification of cod liver oil increases the bioavailability of EPA and DHA by more rapidly incorporating them into TAG-rich lipoproteins. We also found that juice and yogurt were equally effective as LC *n*-3 FA carriers. Furthermore, the present study provides additional evidence that the acute intake of oxidised cod liver oil does not influence the incorporation of LC *n*-3 FA into TAG-rich lipoproteins.

## Supplementary material

For supplementary material accompanying this paper visit http://dx.doi.org/10.1017/jns.2016.34. click here to view supplementary material
