# Supplementary material for: Bioavailability of n-3 fatty acids from n-3-enriched foods and fish oil with different oxidative quality in healthy human subjects: a randomised single-meal cross-over study
Source: J Nutr Sci. 2016 Oct 28;5:e43. doi: 10.1017/jns.2016.34 (PMC5465811; doi:10.1017/jns.2016.34)
Supplement: Supplementary file 1 [file S2048679016000343sup001.docx]

| **Supplementary Table S1.** Fatty acid profile in the test meals | | | | |  |
| --- | --- | --- | --- | --- | --- |
|  | Yogurt meal | Juice meal | Non-oxidized cod liver oil meal | Oxidized cod liver oil meal | Reference meal |
| Fatty acids | mg/100 g | | | | |
| C16:1 (*n*-7) | 120.0 | 127.6 | 127.2 | 121.6 | 19.2 |
| C18:1 (*n*-9) | 2561.5 | 2642.9 | 2629.1 | 2629.4 | 3549.3 |
| C18:1 (*n*-7) | 88.1 | 93.4 | 93.5 | 90.8 | 40.0 |
| C18:2 (*n*-6) | 281.2 | 290.7 | 291.9 | 300.8 | 372.7 |
| C18:3 (*n*-3) | 30.8 | 32.1 | 33.2 | 37.4 | 24.8 |
| C20:1 *(n-9)* | 142.3 | 154.2 | 158.0 | 154.4 | 14.8 |
| C18:4 (*n*-3) | 30.6 | 33.4 | 35.0 | 33.5 | 1.1 |
| C22:1 (*n*-9) | 16.3 | 17.4 | 17.6 | 17.1 | 4.0 |
| C20:4 (*n*-3) | 8.5 | 9.4 | 9.5 | 8.9 | 0.3 |
| C20:5 (*n*-3) | 116.2 | 124.9 | 125.0 | 117.0 | 0.1 |
| C22:5 (*n*-3) | 15.3 | 16.4 | 16.3 | 15.8 | 0.8 |
| C22:6 (*n*-3) | 146.6 | 157.5 | 158.3 | 151.3 | 0.0 |
| ƩSFA | 1327.3 | 1370.9 | 1359.3 | 1331.6 | 1227.0 |
| ƩMUFA | 3397.3 | 3525.3 | 3518.7 | 3516.2 | 4154.6 |
| ƩPUFA | 335.3 | 359.7 | 362.9 | 344.3 | 12.3 |
